# Prospective Audit and Feedback of Targeted Antimicrobials Use at a Tertiary Care Hospital in the United Arab Emirates

**DOI:** 10.3390/antibiotics14030237

**Published:** 2025-02-26

**Authors:** Shabaz Mohiuddin Gulam, Dixon Thomas, Fiaz Ahamed, Danial E. Baker

**Affiliations:** 1 College of Pharmacy, Gulf Medical University, Ajman 4184, United Arab Emirates; dr.dixon@gmu.ac.ae; 2 Clinical Pharmacy Department, Thumbay University Hospital, Ajman 4184, United Arab Emirates; 3 Infection Control Department, Thumbay University Hospital, Ajman 4184, United Arab Emirates; dr.fiaz@thumbayuniversityhospital.com; 4 College of Pharmacy and Pharmaceutical Sciences, Washington State University, Spokane, WA 99202, USA

**Keywords:** pilot study, antimicrobial stewardship, pharmacist-led intervention, United Arab Emirates

## Abstract

**Background/Objectives:** Antimicrobial stewardship programs improve antimicrobial use and help combat antimicrobial resistance. The Infectious Disease Society of America’s (IDSA) recommended core interventions include prospective audit and feedback along with formulary restriction and preauthorization. IDSA recommends any one of these interventions be implemented in acute care hospitals to improve antimicrobial stewardship. The objective of this project was to implement a prospective audit and feedback system using selected antimicrobials at a tertiary care hospital in the United Arab Emirates as the foundation to build an antimicrobial stewardship program. **Results:** A total of 497 patients met the inclusion and exclusion criteria during the study period; the post-intervention group had 260 patients, and the control group had 237 patients. After the implementation of the program, a total of 186 interventions were recommended, and 76% were accepted. The length of stay, length of therapy, and days of therapy were lower in the intervention group compared to the control group (*p* < 0.05). There was no statistically significant difference in clinical outcome measures (e.g., 30-day readmission, 30-day all-cause mortality, 30-day emergency visit with the same infection, and 60-day readmission). **Methods:** This single-center quasi-experimental research was conducted from August 2023 to July 2024. A pharmacist-led prospective audit and feedback system was initiated in February 2024 after review and approval of the medical staff, in addition to formulary restrictions. Data from patients receiving the selected antimicrobial before February 2024 were collected from their charts and related medical records without any intervention; this was used by our control group. After implementation, the hospital pharmacy’s records were evaluated during the night shift to determine whether they met the inclusion criteria. The records of the eligible patients were then evaluated by the clinical pharmacist. In case of antimicrobial inappropriateness, feedback was provided to the prescriber. If the recommendation was not accepted, succeeding reviews and feedback were provided on subsequent days. The effectiveness of the intervention was measured using clinical and antibiotic use measures. **Conclusions:** Implementation of a pilot pharmacist-led antimicrobial stewardship program resulted in modification in antimicrobial use measures (i.e., defined daily doses of targeted antimicrobials and days of antimicrobial therapy) without an increase in length of stay or readmissions or mortality.

## 1. Introduction

Antimicrobial resistance (AMR) has become a global concern due to alarming increasing rates of resistance and the diminishing pipeline of new antibiotics in the market. Consequences of AMR include ineffective antimicrobials resulting in infections becoming difficult or impossible to treat and an increase in infection severity and death. Overuse and irresponsible use of antimicrobials across diverse settings, including clinical settings, have been associated with increasing AMR [1,2].

The United Arab Emirates’ (UAE) healthcare system features modern infrastructure and a diverse, multicultural population [3,4]. While cities like Dubai and Abu Dhabi have advanced medical facilities, smaller emirates face challenges such as limited access to trained antimicrobial stewardship personnel and inadequate integration of electronic medical records [4,5]. Additionally, the dominance of private healthcare and time constraints for clinicians complicate the implementation of stewardship interventions [6]. However, the UAE’s national AMR strategy offers opportunities to strengthen AMS programs through targeted planning, education, and communication efforts [7].

To promote the appropriate use of antimicrobials, many organizations around the globe, like the World Health Organization (WHO), Centers for Disease Control and Prevention (CDC), the Infectious Disease Society of America (IDSA), and Joint Commission International (JCI), recommend the implementation of antimicrobial stewardship programs [8,9,10]. In the United Arab Emirates, the Department of Health Abu Dhabi released guidelines in 2017 to assist healthcare organizations in stewardship programs with the objective of improving antimicrobial prescribing [11].

Various interventions are possible as a part of the antimicrobial stewardship program. Examples of these types of interventions include formulation restriction, prospective audit and feedback, institutional guidelines, clinical pathways, antibiograms, rapid diagnostics, promoting appropriate duration of therapy, and intravenous to oral conversion. An organization can implement one or more of the above interventions as part of its antimicrobial stewardship program [9,11,12].

IDSA recommends hospital antimicrobial stewardship programs implement either a formulary restriction, a prospective audit and feedback program, or both as the foundation of their antimicrobial stewardship program [6]. The decision on which intervention to implement depends on the institution’s resources (e.g., availability of expertise, staffing, and financial resources). The advantages of the formulary restriction process include improved antimicrobial selection prior to or at the time of the medication order, while prospective audit and feedback methods are implemented after the medication order [13].

The prospective audit and feedback process involves a comprehensive review of the antimicrobial order after it has been conducted and requires the prescriber to be contacted either prior to the order being filled or after the patient receives at least one dose of the medication with a recommendation to prescribers on therapy modification. During the prospective audit and feedback process, there is an interaction between the antimicrobial steward and prescriber, which leads to discussion and knowledge transfer, often known as ‘handshake stewardship’ [14,15]. However, reviewing each patient on antibiotics from admission through discharge may be cumbersome and will need huge resources and manpower. To counter this disadvantage of prospective audit and feedback, institutional stewardship programs can determine their own criteria to identify patients that require a more thorough evaluation. This type of selection criteria may limit the program to those patients requiring multiple antimicrobials, those on targeted antimicrobial agents, or those admitted to certain high-risk units (e.g., intensive care units, cardiac care units) [16,17].

The audit of antimicrobials should be performed by practitioners with expertise in the area of infectious disease. Selection of the outcome measures in antimicrobial stewardship programs depends on the type of intervention implemented and the timeframe of evaluation. Outcome measures like resistance patterns need a longer time of monitoring, which will require longer periods of time to determine the impact of the program on resistance rates. Antimicrobial use measures like defined daily doses, days of therapy, and length of therapy are commonly used in antimicrobial stewardship programs alongside clinical outcome measures like mortality, length of stay, readmission rates, and cure rates [9,10,11,16]. Pharmacist-led antimicrobial stewardship programs involving prospective audits and feedback have been reported to improve antimicrobial use [18,19,20,21,22,23]. There is a high use of broad-spectrum antimicrobials in institutional settings [24]. The clinical outcome and antimicrobial use measures are both best needed to quantify the outcomes of the programs [25]. Pharmaceutical Care Network Europe outlines the various categorization of pharmacist interventions in relation to their acceptance [26].

In this study, we have analyzed the effect of pharmacist-led prospective audits and feedback programs on clinical and antimicrobial use measures. We have also analyzed the type of recommendations provided along with their acceptance rates.

## 2. Results

A total of 497 patients met the inclusion and exclusion criteria during the study period. The post-intervention group had 260 patients, and the control group had 237 patients. There was no statistical difference in the groups in terms of age, gender, previous hospitalization in the past 3 months, and number of comorbidities. However, the post-intervention group had 38.1% of patients with a history of antibiotic use in the past 3 months compared to the control group, 26.6% (*p* ≤ 0.05). The classification of patients based on type of infection and other demographics is presented in Table 1.

Among the alerts received from the pharmacy during the post-intervention period of February to July 2024, the data related to 260 patients were included in the analysis. A total of 186 interventions related to prospective audit and feedback were proposed in 144 patients (Table 2). The overall percentage of acceptance and implementation of feedback was 76%. Feedback was neither accepted nor implemented in 45 interventions.

The majority (37.1%) of the interventions were related to the discontinuation of the antimicrobial after empirical initiation of the therapy, as there was either no indication for use or redundancy in the antimicrobial spectrum (Table 3). Changing the antimicrobial to avoid unnecessary broad-spectrum was performed in 42 interventions. Other interventions that were performed include dose optimization (14.5%) and recommending laboratory monitoring (8.6%).

The length of stay, length of therapy, and days of antimicrobial therapy were lower (*p* < 0.05) in the intervention group compared to the control group. There was no statistically significant difference in clinical outcome measures like 30-day readmission, 30-day all-cause mortality, 30-day emergency visits with the same problem, and 60-day readmission (Table 4).

**Figure 1:**
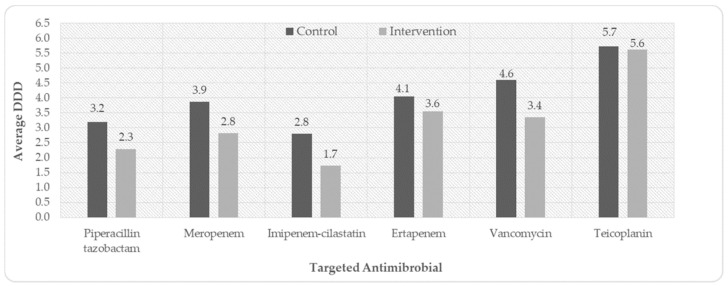
Average defined daily doses of targeted antibiotics.

The average defined daily doses of targeted antimicrobials are presented in Figure 1. With the prospective audit and feedback process, the defined daily doses of the targeted antimicrobials were lower in the intervention group compared to the control group. The antimicrobials for which the defined daily doses increased with the intervention compared to the control group include cefdinir (5 vs. 5.71) and ciprofloxacin (5.5 vs. 7.2).

## 3. Discussion

Our study describes the implementation of a pharmacist-led prospective audit and feedback program as part of an antimicrobial stewardship program at a tertiary care hospital in the United Arab Emirates. The institution already has a formulary restriction intervention, and this pharmacist-led prospective audit and feedback was the additional intervention implemented. The outcome measures of the intervention group were compared to 6 months of pre-intervention patients’ group identified as the control.

Prospective audit and feedback is a resource-intensive intervention as it involves a prospective review of patients on antimicrobials every day [27]. To cater the service to the most appropriate patient group, a list of targeted antibiotics was made, which involved piperacillin–tazobactam, meropenem, imipenem–cilastatin, ertapenem, vancomycin, and teicoplanin, as their use has the scope of improvement [28,29,30].

Alerts from the pharmacy on a daily basis during the night shift helped to identify the patients who had active orders of these targeted antibiotics. We included only adult patients in our study. Neonates, pediatrics, and long-term care patients were excluded to decrease the negative impact of their inclusion on the analysis of outcome measures like defined daily doses and length of stay [31,32].

Patients in the control group were identified from the pharmacy database. While screening the control patients, we excluded all the patients with hospitalization of less than 24 h to reduce the impact of formulary restriction. The number of patients in the intervention group is more than the number of patients in the control group, which may be due to potential seasonal variations because the two evaluation periods are at different times of the year.

In the current study, males comprised a slightly higher proportion of the patient population, with 62.7% in the intervention group compared to 59.9% in the control group. In the UAE, studies show a male predominance in hospital admissions. For instance, a study conducted in Abu Dhabi reported higher male representation in the patient population receiving antibiotics [29,33]. This trend can be attributed to differences in the gender distribution of men and women in the region, with 78% of the population being male [34].

The mean age in both the control and intervention groups is approximately 42 years. This is consistent with studies conducted in the UAE, where adult populations in their 40s and 50s often comprise a larger proportion of hospitalized patients receiving antibiotics [35]. The age distribution reflects the typical demographic profile in the UAE, with a large segment of the population consisting of working-age expatriates [33].

There was a difference in the type of infections in our patients in the intervention and control groups, although the majority of them were suffering from infections of the respiratory tract, genitourinary system, skin soft tissue infection, and intra-abdominal and gastrointestinal tract. These are the infections that are commonly seen in the UAE [36,37].

Our population did not differ in terms of hospitalization in the previous 3 months; however, the intervention group had more patients with a history of antibiotic use. As our criteria of selection were patients’ identification from the alerts from the pharmacy based on the target antibiotic list, hence the patient groups were different.

The acceptance of interventions is crucial for ensuring success in improving antimicrobial prescribing practices, particularly with prospective audit and feedback programs. In this study, the acceptance rate of interventions was 76%, which is comparable to a similar stewardship program implementation in a Veterans Affairs Medical Center in the United States [38]. In similar research, Elrefaei et al. observed an acceptance rate of over 90% in their quaternary care hospital [36]. The reason for this difference may be because of shorter hospitalization in our setting (3 days) when the length of stay in a study by Elrefaei et al. was 16 days. This shorter duration of hospitalization affected the implementation of interventions because of the absence of confirmatory tests. Our setting does not have rapid infectious diagnostic testing. Similar trends were observed in the Middle East, where Khdour et al. reported that 78% of antimicrobial interventions were accepted in their ICU setting [39].

In this study, the majority of the interventions involved the discontinuation of antibiotics after empirical therapy initiation, which was accepted in most cases. This pattern is consistent with other studies in the region [36,39]. More efforts and collaboration are required to increase the acceptance of interventions.

The implementation of prospective audit and feedback intervention at our hospital demonstrated important findings in terms of antibiotic usage and clinical measures. While the clinical outcomes did not have statistically significant differences between the control and intervention groups, the improvements in antibiotic use were promising and aligned with the goals of antimicrobial stewardship.

The clinical measures, including 30-day readmission rates, 30-day emergency visits, 60-day readmission rates, and 30-day all-cause mortality, showed no significant differences between the control and intervention groups. Specifically, the 30-day readmission rate in the intervention group was 7.3% compared to 11.4% in the control group, but this difference did not reach statistical significance (*p* = 0.124). Likewise, the 30-day all-cause mortality rate was comparable between both groups (0.8% vs. 0.4%, *p* = 1.000). These findings are consistent with other studies that report mixed results regarding clinical outcome improvements following the implementation of PAF interventions [29].

Although there was no significant impact on mortality or readmission rates, the slight reductions in these rates suggest that with a longer intervention period or a larger sample size, more pronounced effects might be observed.

One significant outcome was the reduction in the length of hospital stay, which decreased from a median of 4 days in the control group to 3 days in the intervention group (*p* = 0.018). This outcome is in line with findings from previous research, where antimicrobial stewardship interventions have been linked to shorter hospital stays, particularly in high-risk settings such as intensive care units [39]. In the study by Elrefaei et al., incorporating a clinical pharmacist into the weekend antimicrobial stewardship program led to a significant reduction in the length of hospital stay (LOS) from a median of 27.5 days to 16 days (*p* = 0.001) [36]. Reducing hospital stays not only benefits patient recovery but also decreases healthcare costs, emphasizing the broader systemic impact of effective stewardship programs [40].

The program had a notable effect on antibiotic use measures, which were significantly better in the intervention group compared to the control group. The length of therapy was reduced from a median of 9 days to 8 days (*p* < 0.001), and the days of therapy (DOT) were reduced from a median of 5 days to 4 days (*p* = 0.001). This mirrors findings in Shamseddine et al., where ASP interventions significantly reduced days of therapy (DOT) and improved guideline adherence in a large tertiary hospital [37]. Furthermore, Khdour et al. demonstrated the effectiveness of PAF in reducing antibiotic consumption by 24.3% and shortening the duration of therapy from 8 days to 5 days in ICU patients [39].

Reducing the length of therapy and DOT is critical in minimizing the risks associated with prolonged antibiotic use, including the development of antimicrobial resistance (AMR) and adverse drug events [37]. Moreover, this intervention aligns with the goals of antimicrobial stewardship programs worldwide, which aim to optimize antibiotic therapy by reducing unnecessary or prolonged use [41].

Antimicrobial overuse is one of the reasons for the development of resistant microorganisms. There was an overall reduction of 22.5% in the defined daily doses of targeted antibiotics in the intervention period compared to the pre-intervention, showing a reduction in antimicrobial overuse. In the study by Sadeq et al. (2021), piperacillin–tazobactam and meropenem consumption decreased significantly, leading to improved clinical outcomes such as reduced length of hospital stay and cost savings [29]. This is consistent with the findings from Khdour et al. (2017), where the implementation of an ASP in a Palestinian hospital reduced DDD by approximately 24.3%, with significant decreases in ceftriaxone and meropenem usage [39]. The DDDs of non-targeted antimicrobials like cefdinir and ciprofloxacin increased post-intervention. The reason for the increase in DDD of ciprofloxacin may be due to the fact that ciprofloxacin is an antipseudomonal fluoroquinolone [42], and because it was not in the targeted list of antimicrobials, its usage might have increased. The increase in the DDD of cefdinir may be due to intravenous to oral conversion of third-generation cephalosporins. Healthcare facilities opting for pharmacist-led prospective audits and feedback should carefully review their own local practices and select the targeted antimicrobials for their program. The finding that implementation of a stewardship program can lead to an increase in usage of non-restricted antimicrobials must also be considered, like ciprofloxacin in our case.

Although our study demonstrates many important findings, the results of the study should be used by considering the following limitations. The mean length of hospital stay in our institution is smaller compared to the hospitalization periods in similar studies. Hence, findings of clinical outcome measures like 30-day all-cause mortality shall be considered with caution. The majority of our patient population are expatriates who have the tendency to go back to their home country to avail treatment. Hence, clinical outcome measures may not represent a true reflection. We reported the defined daily dose as an average. We did not take into account the time from the recommendation and the prescribers’ action, which can affect the implementation of the intervention. Our facility does not offer rapid diagnostic microbiology testing; however, its implementation can increase the rates of acceptance of interventions.

## 4. Methodology

### 4.1. Design

This single-center quasi-experimental research was conducted at a tertiary care university hospital in the northern emirate of the United Arab Emirates. The data were collected from August 2023 to July 2024. Additional pharmacist led prospective audit and feedback intervention was initiated from February 2024 apart from formulary restriction. This study was conducted with the approval of the institutional review board after review and approval of the medical staff.

### 4.2. Intervention

**Figure 2:**
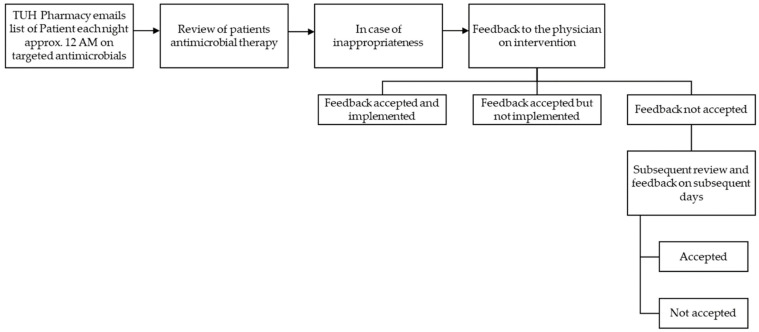
Prospective audit and feedback process adopted.

The intervention was a prospective audit of antimicrobial prescriptions among inpatients in February 2024. The pharmacy department emails a list of patients on targeted antimicrobials each night (12 AM). As it is difficult to review all inpatients who were prescribed antibiotics, we have carefully selected the targeted list to include broad-spectrum and reserved antimicrobials [10]. These antimicrobials in the targeted list were piperacillin–tazobactam, meropenem, imipenem–cilastatin, ertapenem, vancomycin, and teicoplanin [24]. A comprehensive review of patients’ antimicrobial therapy was conducted daily, taking into account patients’ clinical status, organ functions, laboratory and radiological information, and antibiotic history. We followed the checklist for antibiotic review during the prospective audit and feedback, like the selection of antibiotics, dose optimization, intravenous to oral conversion, drug interactions, antibiotic streamlining, laboratory monitoring, discontinuation of antibiotics if not required, and appropriateness of duration. In case of antimicrobial inappropriateness, feedback was provided to the physician on intervention. The feedback was provided by meeting with the prescribers following a handshake approach. When the prescriber was off duty, feedback was communicated via phone call or through the ward general practitioner. If intervention is not accepted or accepted but not implemented, subsequent review and feedback on subsequent days are conducted. The prospective audit and feedback process adopted is detailed in Figure 2 [25]. All the patients alerted by the pharmacy were included in the analysis except long-term care, neonates, and pediatric patients. These patient categories are excluded because their inclusion could have negatively impacted the outcomes monitored in the study. The antimicrobial use measure of defined daily doses cannot be applied to pediatric and neonates [25]. Similarly, when applying the length of stay as an outcome measure, combining acute care and long-term care patient groups is not appropriate.

### 4.3. Control

From the pharmacy database, the list of patients who were prescribed the targeted antimicrobials was collected from August 2023 to January 2024. Long-term care, neonates, and pediatric patients were excluded from the analysis to match the exclusion criteria of the intervention group. Additionally, patients who were hospitalized for less than 24 h were excluded from the analysis to limit the effect of formulary restriction, which was already in place.

### 4.4. Outcome Measures

Clinical outcome measures and antimicrobial use measures were used to analyze the difference between pre- and post-intervention. The clinical outcome measures analyzed were the length of stay, 30-day readmission, 60-day readmission, 30-day emergency visits for the same condition, and 30-day all-cause mortality. The antimicrobial use measures analyzed were defined as daily doses, days of therapy, and length of therapy. The outcome of individual patient intervention was measured as intervention accepted and implemented, accepted but not implemented, and not accepted, adopted from the classifications of interventions from the Pharmaceutical Care Network Europe [26].

### 4.5. Statistical Analysis

The statistical analysis of data was conducted using SPSS version 26 (IBM Corporation: Armonk, NY, USA). The chi-square test was used to analyze the differences between categorical variables. The Kolmogorov–Smirnov Z test was applied for the continuous variable to analyze the distribution of data. An independent *t*-test was used to analyze the differences in the parametric data, and the Mann–Whitney U test was applied to analyze the differences in the non-parametric data. A pivot table in Microsoft Excel was used to analyze the types and outcomes of interventions.

## 5. Conclusions

Overall, the implementation of pharmacist-led prospective audits and feedback resulted in reductions in antibiotic use measures like defined daily doses of targeted antibiotics and days of antibiotic therapy without an increase in length of stay readmissions or mortality. Prospective audits and feedback remain powerful tools in improving the appropriateness of antimicrobial prescriptions without taking away the autonomy of the prescribers. The program will be continued at the facility to improve antibiotic use. Healthcare facilities implementing antimicrobial stewardship programs should incorporate pharmacist-led prospective audits and feedback as part of a comprehensive strategy alongside other interventions. Evaluating the long-term outcomes of this approach can further enhance its effectiveness and overall impact on antimicrobial use and patient care.

## Figures and Tables

**Table 1:** Demographic characteristics of the patients.

Demographic Characteristics	Control Group Number (%) (N = 237)	Intervention Group Number (%) (N = 260)	*p*-Value
Mean age ± standard deviation	41.8 ± 14.4	42.2 ± 15.5	0.72
**Gender**			
Male	142 (59.9)	163 (62.7)	0.58
Female	95 (40.1)	97 (37.7)	
**Previous hospitalization within 3 months**			
**Yes**	25 (10.5)	35 (13.5)	0.36
**No**	212 (89.5)	225 (86.5)	
**Previous antibiotic use within 3 months**			
Yes	63 (26.6)	99 (38.1)	0.007
No	174 (73.4)	161 (61.9)	
**No of comorbidities**			
0	127 (53.6)	143 (55)	0.85
1–2	80 (33.8)	80 (30.8)	
3–4	24 (10.1)	31 (11.9)	
More than 5	6 (2.5)	6 (2.3)	
**Type of infection**			
Genitourinary infection	68 (28.7)	70 (26.9)	0.01
Infection of the central nervous system	7 (3.0)	2 (0.8)	
Intra-abdominal infection/Gastrointestinal infection	58 (24.5)	46 (17.7)	
Respiratory infection	44 (18.6)	75 (28.8)	
Sepsis/unknown source of infection	25 (10.5)	39 (15)	
Skin and soft tissue infection/bone and joint infection	35 (14.8)	28 (10.8)	

**Table 2:** Outcome of interventions.

Outcome of Interventions	Number (N = 186)	Percentage
**Acceptance of intervention**		
Accepted and implemented	119	64
Accepted but not implemented	14	7.5
Not accepted	53	28.5
**Was intervention accepted on subsequent days?**		
Yes	22	11.8
No	45	24.1

**Table 3:** Type of Interventions.

Type of Intervention	Number (N = 186)	Percentage
Addition of antibiotic	5	2.7
Changing of antibiotic	42	22.5
De-escalation of antibiotic therapy	14	7.5
Discontinue antibiotics	66	35.5
Eliminate redundant therapy	47	25.3
No indication for the use of antibiotic	19	10.2
Dose optimization	27	14.52
Decrease dose	21	11.3
Increase dose	6	3.2
Escalation of antibiotic	8	4.3
Optimize the duration of antibiotic	6	3.23
IV to Oral therapy	2	1.08
Recommend lab monitoring	16	8.6

**Table 4:** Outcome measures.

	Control Group Number (%) (N = 237)	Intervention Group Number (%) (N = 260)	*p*-Value
*Clinical measures*			
30-day readmission	27 (11.4)	19 (7.3)	0.124
30-day emergency visit with the same problem	15 (6.3)	21 (8.1)	0.492
60-day readmission	21 (4.6)	10 (3.8)	0.664
30-day all-cause mortality	1 (0.4)	2 (0.8)	1.000
Length of stay	4 (3–6)	3 (2.5–5)	0.018
*Antibiotic use measure*			
Length of therapy	9 (8–11)	8 (7–10)	<0.001
Days of therapy	5 (3–9)	4 (3–6.5)	0.001

## Data Availability

In case of any data, the corresponding authors can be contacted at dr.shabaz@gmu.ac.ae.
